# High‐Performance Liquid Chromatography Method Optimization and Validation for Potassium Bromate Detection in Bread Using a Quality‐by‐Design Approach

**DOI:** 10.1155/jamc/9966003

**Published:** 2026-05-20

**Authors:** Tesfamichael Gebretsadikan, Mebrahtom Gebrelibanos Hiben, Kalayou Hiluf Gebremedhin, Mulaynesh Zebrehe, Hayelom Fisseha, Teklu Hailu Abay

**Affiliations:** ^1^ School of Pharmacy, College of Health Sciences, Mekelle University, P.O. Box 1871, Mekelle, Tigray, Ethiopia, mu.edu.et; ^2^ Department of Chemistry, College of Natural and Computational Sciences, Mekelle University, P.O. Box 231, Mekelle, Tigray, Ethiopia, mu.edu.et

**Keywords:** central composite design, HPLC, method development and validation, potassium bromate, processed bread, quality by design

## Abstract

**Background:**

Potassium bromate (KBrO_3_) is a flour additive that strengthens dough and increases bread volume. However, the International Agency for Research on Cancer (IARC) has labeled it as a possible human carcinogen. Despite being banned in many countries, KBrO_3_ is still found in bread, particularly in resource‐limited settings like Ethiopia, which can pose serious health risks.

**Objective of Study:**

The goal of this study is to develop and validate a sensitive, selective, and accurate high‐performance liquid chromatography (HPLC) method for detecting and measuring KBrO_3_ in bread using a quality by design (QbD) approach.

**Methodology:**

A reverse‐phase HPLC method was developed and optimized using the central composite design (CCD) expert. Chromatographic analysis was performed on a C18 column with a mobile phase consisting of acetonitrile and phosphate buffer. The method was validated following the ICH Q2(R1) guidelines. Fifteen commercial bread samples were collected from each subcity of Mekelle and analyzed using the developed method.

**Results:**

The developed method demonstrated excellent linearity, precision, and accuracy, with recovery rates between 96% and 99%. The limit of detection (LOD) was 0.015 mg/kg. Bromate residues were detected in all bread samples, with concentrations ranging from 4.5 to 9.15 mg/kg, which is significantly higher than the WHO/FDA safety limit of 0.02 mg/kg.

**Conclusion:**

The validated HPLC method provides a reliable and accurate tool for routine monitoring of KBrO_3_ in bread. The results highlight significant public health risks associated with excessive bromate use in local bread production and call attention to the urgent need for strengthened regulatory controls and enforcement of food safety standards in Ethiopia.

## 1. Introduction

Bread, one of the most universal foods, is mostly wheat‐based and is a staple in many countries. The average global consumption of bread ranges from 59 to 70 kg per year per capita [1]. The use of bread as a basic dietary item dates back to the Neolithic era. Bread is an essential cereal product in human nutrition, providing as much as 50%–90% of total caloric and protein intake in Ethiopia. It is most commonly consumed at home, in hotels, and in restaurants [2, 3]. To stay competitive, the food and manufacturing industries use a range of bread ingredients. Among the fundamental components are flavors, sugars, and table salt. Additionally, bakers have been using large amounts of potassium bromate (KBrO_3_) to enhance the texture and loaf volume of their breads [4]. KBrO_3_ is inexpensive and is probably the most effective oxidizing agent, which explains its frequent use by flour millers and bakers worldwide [3].

It acts as a slow oxidizing agent throughout fermentation, proofing, and baking, affecting the structure and rheological properties of the dough. KBrO_3_ oxidizes the sulfhydryl groups of flour’s gluten proteins, making the dough less extensible and more elastic, thereby enabling it to retain the carbon dioxide gas produced by yeast during leavening [5]. As a result, many bakeries use KBrO_3_ as an additive to assist in the raising process and to produce a texture in the finished product that is appealing to the public.

However, the International Agency for Research on Cancer (IARC) has classified KBrO_3_ as a category 2B compound, indicating that it is possibly carcinogenic to humans. Animal studies have shown an increased risk of tumors, particularly in the kidney and thyroid [6, 7]. KBrO_3_ has been associated with both immediate and long‐term health effects. Abdominal pain, diarrhea, irritation of the upper aerodigestive mucosa, and vomiting are among the short‐term symptoms of KBrO_3_ exposure [8, 9]. In human blood, KBrO_3_‐derived free radicals can cause cancer and nephrotoxicity. Similarly, it induces mesotheliomas, thyroid follicular cell tumors, and renal cell tumors in rats. Chronic consumption of potassium bromates damages the tissues of the kidneys and central nervous system (CNS) [10]. Moreover, KBrO_3_ adversely affects the nutritional quality of bread by degrading vitamins A2, B1, B2, E, and niacin [9]. Consequently, several organizations, agencies, and countries, such as the European Union, Canada, and Brazil, have banned its use as a flour additive, while countries like China, India, and Japan have established maximum acceptable doses for finished baked products [11].

Despite existing regulations, concerns remain about the presence of KBrO_3_ in bread, making it essential to conduct thorough analyses to ensure consumer safety. Studies conducted in Addis Ababa, Ethiopia, have detected KBrO_3_ in bread samples from various bakeries, but there is no official documentation mentioning a ban on KBrO_3_ in Ethiopia [2]. Furthermore, in Mekelle, no study has yet been conducted regarding the presence and concentrations of KBrO_3_ in bread, creating a notable gap in knowledge regarding its health risks and use. Regulatory bodies such as the WHO and FDA have called for its ban or restricted use in food. Detecting KBrO_3_ in food products has been challenging due to limitations in existing methods, such as poor sensitivity, long analysis times, and the need for highly skilled operators. In this study, we aimed to address these issues by applying a quality by design (QbD) approach with a central composite design (CCD) to develop a robust and reproducible method for detecting KBrO_3_ at minute concentrations.

## 2. Materials and Methods

### 2.1. Study Area

The study was conducted in Mekelle City, the capital of the Tigray Region in northern Ethiopia. Mekelle is a vibrant urban center with a rich historical and cultural background and a population exceeding 612,000. The high population density contributes to substantial bread production and consumption in the city. Laboratory analyses were carried out at the Mekelle University, College of Health Sciences, School of Pharmacy laboratories.

### 2.2. Study Design

The study was designed as a laboratory‐based observational study aimed at optimizing and validating a high‐performance liquid chromatography (HPLC) method for the detection and quantification of KBrO_3_ in bread. The QbD approach was utilized for the development and optimization of the method, ensuring a systematic evaluation of critical method parameters (CMPs) such as mobile phase (MP) composition and buffer pH. The study focused on assessing the presence and concentration of KBrO_3_ in samples collected from different urban subcities, while also evaluating variations in bread quality across locations.

#### 2.2.1. Sampling

A stratified random sampling technique was employed to ensure representative sampling across subcities. A total of 15 bread samples were collected from each subcity, covering major bakeries and local markets. Stratification was based on geographic location and bakery type to account for potential variability in flour sources and baking practices. All samples were properly labeled, stored in airtight containers, and transported to the laboratory under controlled conditions to maintain sample integrity until analysis.

#### 2.2.2. Chemicals and Reagents

HPLC‐grade acetonitrile (ACN) (Loba Chemie Pvt. Ltd., Mumbai, India), potassium dihydrogen phosphate (KH_2_PO_4_) (Loba Chemie Pvt. Ltd., Mumbai, India), and dipotassium hydrogen phosphate (K_2_HPO_4_) (Loba Chemie Pvt. Ltd., Mumbai, India) were used in the study. Double‐distilled water was purchased from Jourilabs, Addis Ababa, Ethiopia. A 100% pure KBrO_3_ reference standard was obtained from Addis Pharmaceutical Factory, Adigrat, Ethiopia. Additionally, uracil obtained from EFDA was also used as an unretained marker.

#### 2.2.3. Instrumentation

Liquid chromatography analyses were carried out using an Agilent 1260 Infinity Series HPLC system (Agilent Technologies, Waldbronn, Germany), equipped with an Infinity Binary Pump (G1312B), High Performance Degasser (G4225A), High Performance Autosampler (G1367E), Thermostat Column Compartment (G1316C), and Photodiode Array Detector (G4212B). Chromatographic separation was performed on a C18 column (250 × 4.6 mm, 5 μm) (Tokyo, Japan). A digital pH meter (Adwa Instruments, Bucharest, Romania), an analytical balance (Mettler Toledo, Greifensee, Switzerland), and various volumetric flasks were also used.

### 2.3. Preparation of Working Standard Solution

Five milligrams of KBrO_3_ standard were weighed and transferred to a 100 mL volumetric flask. The mixture was dissolved using 40 mL of the MP and diluted to the mark with the same solvent to obtain a standard solution of 0.05 mg/mL KBrO_3_. Standard solutions were freshly prepared on the day of analysis and filtered using a 0.45 μm nylon membrane filter before use [12].

### 2.4. Sample Preparation

The bread was collected and oven‐dried at a controlled temperature until constant weight and then powdered using a pestle and mortar. From the powdered sample, 10 mg was weighed and transferred into a test tube. Ten milliliters of distilled water were added, and the mixture was shaken and allowed to stand for 20 min. A 5 mL volume was then decanted and diluted in 100 mL of distilled water. Finally, 1 mL of the resulting solution was transferred to an autosampler vial for HPLC analysis.

### 2.5. Preliminary Optimization of Chromatographic Conditions

Initially, several trial‐and‐error experiments were conducted using different chromatographic parameters to develop a suitable HPLC method for the determination of KBrO_3_. ACN and K_2_HPO_4_ were selected as the MP components based on the physicochemical properties of the analyte and supporting literature. These preliminary trials aimed to identify favorable chromatographic conditions.

### 2.6. QbD‐Based Method Optimization

#### 2.6.1. Defining the Analytical Target Profile (ATP)

The goal was to develop and validate a robust and sensitive reversed‐phase HPLC method for the quantitative determination of trace levels of residual KBrO_3_ in bread samples, ensuring consumer safety and regulatory compliance.

#### 2.6.2. Selection of CMPs and Critical Quality Attributes (CQAs)

CQAs are the key measurable attributes that directly affect the quality, accuracy, and precision of the results. Retention time, theoretical plate number, and tailing factor were identified as CQAs for the detection of KBrO_3_. These parameters reflect the method’s ability to separate, detect, and quantify KBrO_3_ in bread samples accurately and precisely. Based on a comprehensive literature review and preliminary experiments, the MP composition (specifically, the ratio of ACN to phosphate buffer) and buffer pH were selected as CMPs.

CCD was employed as a design of experiment (DoE) to optimize these CMPs and understand their effect on the CQAs, enabling evaluation of both main effects and interaction effects between variables. This approach provided a scientific and efficient means to explore the method’s design space and determine optimal conditions [13]. The DoE results facilitated the identification of a robust set of chromatographic conditions that consistently met all predefined acceptance criteria, including sharp peaks, sufficient theoretical plates, and acceptable retention times [14]. Surface plots were employed to study the interaction and quadratic effects of MP ratio (60%–70% ACN) and buffer pH (6.5–7.0) on retention time (R1), theoretical plate number (R2), and asymmetry factor (R3). A total of nine runs were generated by the CCD, and their responses were analyzed using Design Expert software Version 11.1.2.

### 2.7. Data Analysis Tools

Design Expert software Version 11.1.2 was used to optimize chromatographic parameters and evaluate their effects on key responses. In addition, a one‐way analysis of variance (ANOVA) was performed to compare the levels of KBrO_3_ across bread samples from different locations, helping to assess whether differences observed between sampling areas were statistically significant.

## 3. Results

### 3.1. Preliminary Optimization of Chromatographic Conditions

**FIGURE 1:**
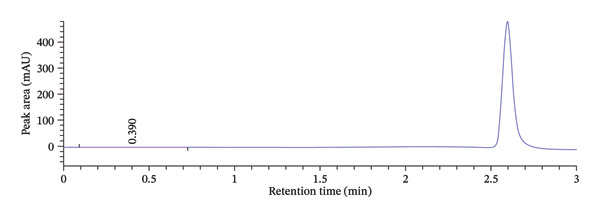
Initial chromatogram prior to central composite design optimization.

As presented in Table 1, some of the tested conditions produced acceptable results in terms of retention time and theoretical plate number. However, the resulting peaks exhibited poor symmetry and significant tailing, which are undesirable for quantitative analysis. This is further illustrated in Figure 1. Therefore, although the initial conditions were promising, further optimization was required to improve peak shape and overall method performance.

**TABLE 1:** Initial trial‐and‐error chromatographic conditions for the determination of potassium bromate and their effects on retention time, theoretical plates, and peak symmetry.

Method parameter	Value	Response			
		No. of theoretical plate	Retention time	Tailing factor	Asymmetry factor
pH	6				
Mobile phase	60%:40% ACN, phosphate buffer				
Flow rate	0.8 mL/min	**3400**	**2.59**	**0.8**	**0.72**
Wave length	210 nm				

*Note:* is to be more visible.

### 3.2. QBD‐Based Method Optimization

**FIGURE 2:**
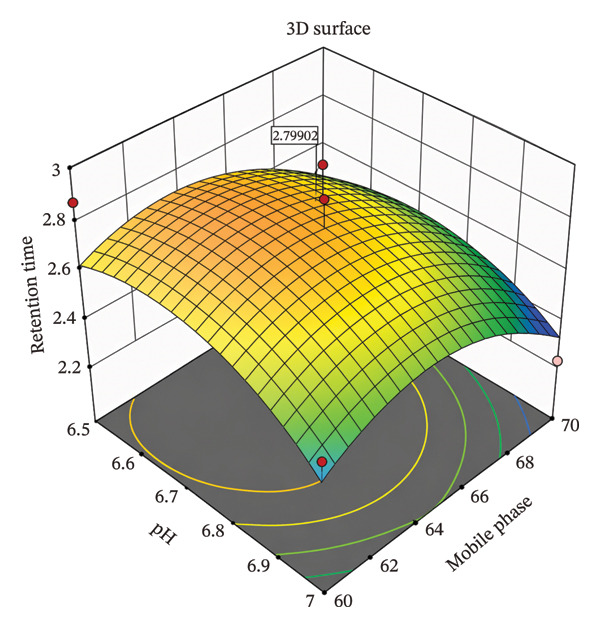
3D surface plot for the effect of pH and mobile phase on retention time of potassium bromate using a central composite design.

**FIGURE 3:**
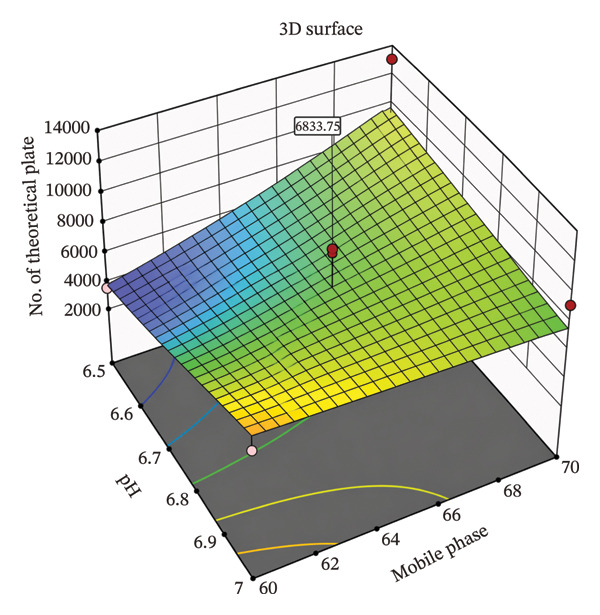
3D surface plot for effects of combination on number of theoretical plates of potassium bromate using central composite design.

**FIGURE 4:**
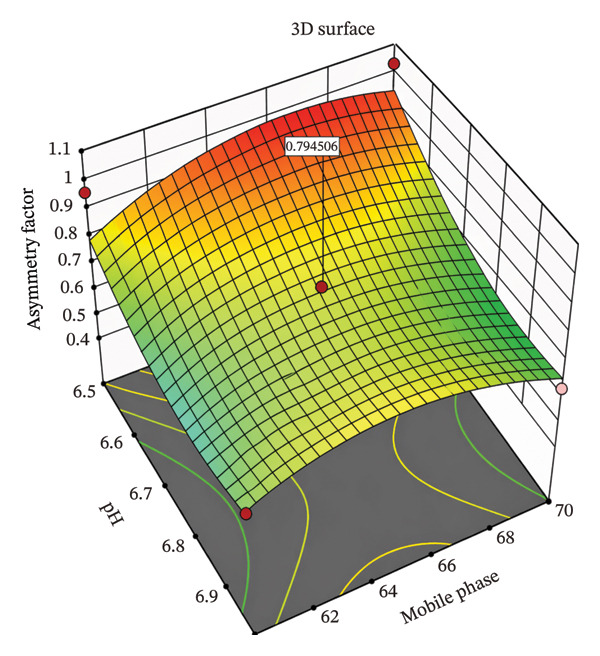
3D surface plot for effects of combination on asymmetry factor of potassium bromate using central composite design.

A comprehensive literature review and preliminary experiments were conducted to select the MP composition specifically, the ratio of ACN to phosphate buffer and the buffer pH as CMPs, with their high, central, and low values provided in Table 2. CCD was employed as a DoEs to optimize these CMPs and evaluate their effects on the CQAs. A total of nine runs were generated by the CCD (Table 3), and their responses were analyzed using Design Expert software Version 11.1.2. Further optimization was demonstrated using a three‐dimensional (3D) surface response design based on the CCD approach, as illustrated in Figures 2, 3, 4.

**TABLE 2:** Coded values for independent variables (CMPs).

Factor	Low value (−1)	Center value (0)	High value (+1)
pH buffer	6	6.5	7
Acetonitrile/buffer	60:40	65:35	70:30

**TABLE 3:** Optimization of parameters for analysis of potassium bromate using CCD.

Run	Factor 1 pH	Factor 2 MP	Response 1 retention time	Response 2 theoretical plate	Response 3 asymmetry factor
1	6	65	2.4	3736	0.95
2	7.00	60	2.9	3684	0.97
3	6	70	2.3	5147	0.4
4	6.5	70	2.3	3425	0.54
5	7.00	65	2.41	13,097	1.03
6	6.50	60	2.9	9502	0.79
7	6.5	65	2.5	9640	0.72
8	6.5	60	2.9	9778	0.7
9	7.00	70	2.2	9296	0.64

Moreover, the interaction and quadratic effect of these two factors (pH and MP composition) can be explained using a second‐order model as shown in the following equations:

(1) Y=β0+β1A+β2B+β12B+β11A2+β22B2,

where *Y* is the measured response, which is either retention time, theoretical plate, or asymmetric factor), *β*_0_ is an overall mean response or intercept constant, *β*_1_ to *β*_22_ are regression model coefficients from experimental runs of the observed experimental value of *Y*. *A* is the effect of pH buffer, and *B* is MP effect (ratio of acetonitrile/buffer solution). *AB*, *A* ^2^, and *B* ^2^ are interactions between *A* and *B* and quadratic effects of *A* and *B*, respectively.

#### 3.2.1. Effects on Retention Time (Y1)

The effects of pH and MP composition on retention time (*Y*_1_) are shown in Figure 2 and explored by Equation (2):

(2) Y1=−111.00727.4760.6990.0382.2410.0075+A+B+AB‐‐A2−B2.

As shown in Figure 2 and Equation (2), the retention time initially increases sharply with pH (β1 is large positive) and slightly with MP composition. The positive interaction term (*A* and *B*) shows a mild synergistic effect when both factors increase. On the other hand, the negative quadratic terms indicate a peak retention time at an optimal pH, beyond which retention decreases. In this case, the MP composition shows minor curvature, implies less effect on retention time.

#### 3.2.2. Effects on Theoretical Plate (Y2)

Figure 3 and Equation (3) show the effect of pH and MP composition on the number of theoretical plates (*Y*_2_)

(3) Y2=−5063.356754.89975.77611.103604+A+B−AB−A2−B2.

As shown in Figure 3 and Equation (3), the number of theoretical plates increases as increasing the pH of buffer solution and MP. The negative quadratic terms indicate efficiency rises up to an optimum point and then declines. This interaction term shows a slight opposite effect at high levels of both factors.

#### 3.2.3. Effects on Asymmetric Factor (Y3)

The effect of pH and MP composition on peak asymmetry (*Y*_3_) is shown in Figure 4 and Equation (4).

(4) Y3=63.721126.11590.80230.02802.04670.0047−A+B−AB+A2−B2.

As shown in both Figure 4 and Equation (4), the β_1_ negative coefficient (−26.12) suggests that as the pH of buffer (*A*) increases, the value of the asymmetry factor decreases sharply, demonstrating the strong influences of pH peak shape. On the other hand, there is a slight increase in asymmetry value as the percentage of acetonitrile increases from 60% to 70%. A positive quadratic term for pH suggests an optimum beyond which asymmetry worsens. MP has a mild, mostly linear impact with minimal curvature.

### 3.3. Optimized Chromatographic Conditions

The optimized conditions were obtained by studying all responses for the given set of buffer pH (6.5–7) and percentage of acetonitrile (60%–70%) using Design Expert software Version 11.1.2, as shown in Table 4. This was illustrated through 3D surface plots showing the interaction between factors on the responses (Figures 2, 3, 4). Based on these results, the optimized HPLC method was identified with an MP consisting of 65% acetonitrile and 35% dipotassium phosphate buffer at pH 6.75. Chromatographic separation was achieved at a flow rate of 0.8 mL/min, with detection at 210 nm and a column temperature of 30°C using a C18 column (250 × 4.6 mm, 5 μm).

**TABLE 4:** Optimized chromatographic conditions.

Method parameters	Optimized value
Mobile phase type	Acetonitrile and hydrogen phosphate
Mobile phase composition	ACN; buffer (65:35)
pH of the buffer	6.75
Flow rate	0.8 mL/min
Temperature	30°C
Column	C 18 (4.6 mm × 5 µm × 25 cm)
Detector wavelength	210 nm

**FIGURE 5:**
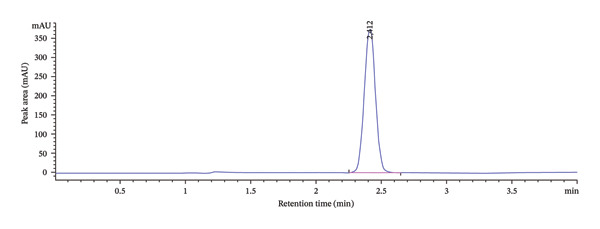
Typical chromatogram obtained with the optimized method.

The method provided a retention time of 2.41 min, high column efficiency with 8706 theoretical plates, and acceptable peak symmetry (asymmetry factor = 0.95), confirming the method’s robustness and suitability for accurate analysis. The developed chromatographic peak under the optimized conditions is presented in Table 4 and further illustrated in Figure 5.

**FIGURE 6:**
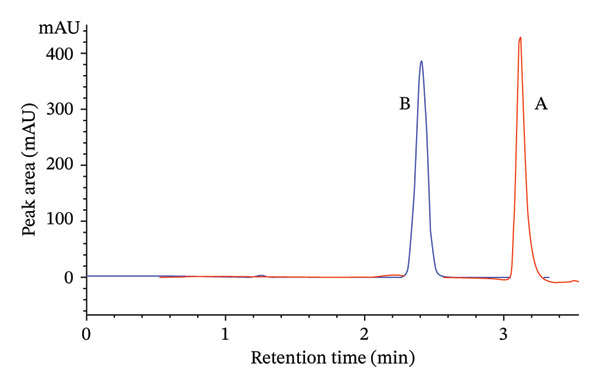
Overlays of chromatograms before (A) and after optimization (B).

The peak A on the overlaid chromatogram (Figure 6) represents the results from our initial trial‐and‐error method, where the retention time was longer, and the peak appeared asymmetrical with significant tailing and a low theoretical plate count, indicating poor separation and inefficiency. In contrast, Peak B, obtained after applying CCD‐based optimization, is sharper and more symmetrical, with a shorter retention time, reduced tailing, and a higher plate count, reflecting a more efficient and reliable method.

These improvements demonstrate the success of the QbD approach in optimizing chromatographic performance. The refined method not only reduced analysis time but also enhanced column efficiency and sensitivity, making it suitable for routine quality control. The optimization provided consistent and reproducible chromatographic behavior, confirming the robustness of the method under varied laboratory conditions.

### 3.4. System Suitability

System suitability is essential for the assurance of the quality performance of chromatographic systems. According to USP, the recommended tailing factor is 0.8–1.2, the asymmetry factor is 0.8–1.2, and the number of theoretical plates is not less than 2000 in method development to accurately quantify and qualify a given chromatogram [15]. Six consecutive injections of the standard solution showed acceptable retention time, theoretical plate count, and asymmetry factor for the product under study, which indicates a good system for the analysis of the sample. Results of the system suitability studies are summarized in Table 5. The void time (*t*_0_) (1.5 min) was determined using uracil as an unretained marker, and the calculated capacity factor (*k* ^′^) for bromate indicated limited but sufficient retention on the C18 column. While the *k* ^′^ (0.606) value suggests weak retention, the chromatographic system provided acceptable peak shape, reproducibility, and resolution without interference from the sample matrix.

**TABLE 5:** System suitability parameters.

Parameter	Obtained values ± %RSD
Asymmetry factor	0.87 ± 0.05
Number of theoretical plates (*N*)	8706 ± 0.02
Retention time	2.41 ± 0.045
Void time (*t*_0_)	1.5 min
Capacity factor (*k* ^′^)	0.606

### 3.5. Method Validation as per ICH Q2 (R1)

The developed HPLC method was validated for its linearity, precision, specificity, accuracy, and robustness following the International Conference on Harmonization Q2 (R1) guidelines [16].

#### 3.5.1. Linearity

**FIGURE 7:**
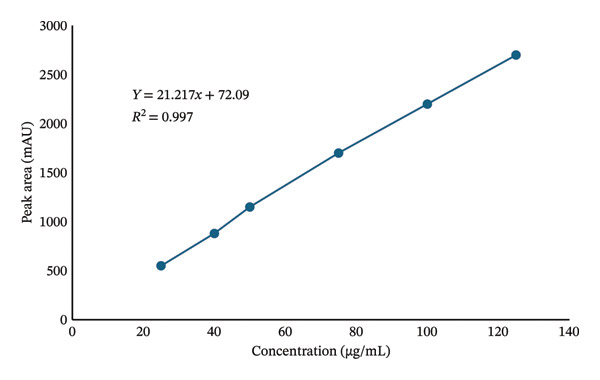
Calibration curve showing the linearity of the developed method.

A series of KBrO_3_ standard solutions (25–125 μg/mL) was prepared and injected in triplicate. A calibration curve was plotted using peak area versus concentration as shown in Figure 7. The method showed satisfactory linearity in the range of 25–125 μg/mL with a correlation coefficient (*R* ^2^) of 0.997, indicating a strong linear relationship.

#### 3.5.2. Accuracy (Recovery)

Known amounts of KBrO_3_ standard were spiked into previously analyzed bread samples at three levels (80%, 100%, and 120% of 5 mg). Each level was analyzed in triplicate. The mean percent recovery (Table 6) ranged from 98.6% to 99.6%, indicating that the method is accurate and free from matrix interference.

**TABLE 6:** Recovery of potassium bromate.

Assay level (%)	Sample added (mg)	Standard added (mg)	Total amount (mg)	Amount recovered	% recovered spiked amount ±SD (*n* = 3)
80	5	4	9	4.02 ± 0.01	99.5 ± 0.24
100	5	5	10	5.02 ± 0.07	99.6 ± 0.01
120	5	6	11	6.08 ± 0.03	98.6 ± 0.6

#### 3.5.3. Precision

The precision of the proposed method was evaluated by repeatability and intraday and interday precision. The %RSD values of repeatability, intraday, and interday precision were found to be within the range of 0.91%, 0.43%, and 1.02%, respectively. The results (Table 7) showed that the mean %RSD values were less than 2%, indicating the good precision of the developed method for determinations of the KBrO_3_.

**TABLE 7:** Results for repeatability, intraday, and interday precision.

Drugs	Repeatability (*n* = 6)		Intraday precision (*n* = 12)		Interday precision (*n* = 18)	
	Mean peak area	%RSD	Mean peak area	%RSD	Mean peak area	%RSD
Potassium bromate	2336.33	0.91	2345.90	0.43	2329.49	1.02

#### 3.5.4. Limit of Detection (LOD) and Limit of Quantification (LOQ)

**FIGURE 8:**
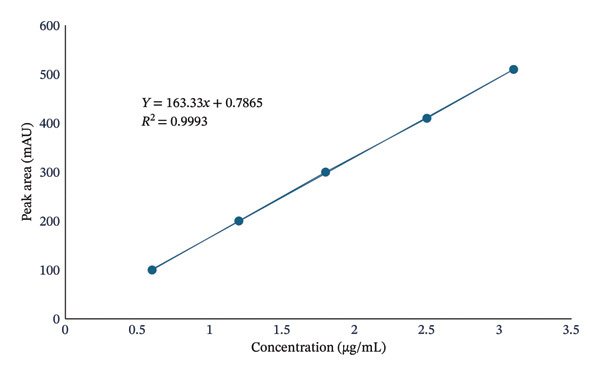
Calibration curve showing the LOD and LOQ of the developed method.

To determine the LOD and LOQ, a separate calibration curve (Figure 8) was constructed using five concentration levels (25%, 50%, 75%, 100%, and 125%), where 100% corresponded to 10% of the lowest concentration from the linearity study. LOD and LOQ were calculated based on the standard deviation of the *y*‐intercept (*σ*) and the slope (*S*) using standard Equations (5) and (6), respectively. The results obtained for LOD and LOQ were 0.015 and 0.04 μg/mL, respectively. These values demonstrate the high sensitivity of the method, capable of detecting KBrO_3_ at levels below WHO limits (0.025 ppm) [2], making it suitable for regulatory and food safety applications.

(5) LOD=3.3×σS,

(6) LOQ=10×σS,

#### 3.5.5. Robustness

The robustness of the method was evaluated by deliberately varying method parameters within a small range, including flow rate (±0.1 mL/min), MP composition (±2%), and pH (±1 unit). No significant changes in retention time or peak shape were observed. The relative standard deviation (RSD) values remained below 2%, confirming the method’s robustness.

#### 3.5.6. Specificity

**FIGURE 9:**
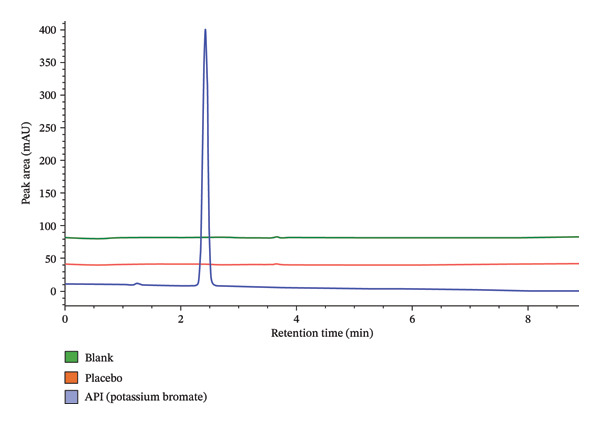
Chromatogram showing specificity of the developed method.

Representative chromatograms of the blank matrix, standard solution, and placebo are shown in Figure 9. No interfering peaks were observed at the retention time of bromate in the blank matrix, confirming the specificity of the method.

### 3.6. Application of the Method in Real Sample

**FIGURE 10:**
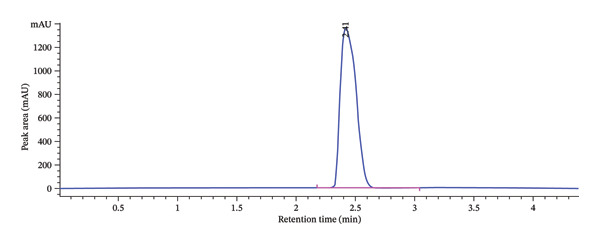
Representative chromatogram of a bread sample collected from the Adihaki Subcity.

In the present study, a total of 15 bread samples were collected from each area of Mekelle City, namely Kedamay Weyane, Hawelti, Hadnet, Adihaki, and Ayder. The determination of KBrO_3_ content in these samples was performed using a robust HPLC method developed based on the QbD approach. The average concentrations of KBrO_3_ detected in bread samples from the different areas were 9.15 mg/kg in Kedamay Weyane, 8.12 mg/kg in Hawelti, 6.5 mg/kg in Hadnet, 4.5 mg/kg in Adihaki, and 7.8 mg/kg in Ayder, as presented in Table 8. All analyzed samples tested positive for KBrO_3_, with levels significantly above the permissible limits set by the WHO and the FDA, both of which recommend a zero‐tolerance policy for KBrO_3_ in baked goods due to its carcinogenic potential. Statistical analysis using one‐way ANOVA showed significant differences (*p* < 0.05) among sampling areas, suggesting varying bakery practices. A representative chromatogram showing good peak shape and symmetry obtained from Adhaki Subcity is presented in Figure 10.

**TABLE 8:** Potassium bromate levels in bread samples.

Area	Amount found (mg/kg)	WHO/FDA limit (mg/kg)	*p* value
Kedamay Weyane	9.15 ± 0.05	0.02	**3.24 × 10** ^**−**^ **^13^**
Hawelti	8.11 ± 0.08	0.02	—
Hadnet	6.50 ± 0.10	0.02	—
Adihaki	4.50 ± 0.057	0.02	—
Ayder	7.80 ± 0.09	0.02	—

*Note:* it is to give emphasize and be more visible.

## 4. Discussion

The present study successfully developed and validated an HPLC method for the detection of KBrO_3_ in bread using a QbD approach. The QbD‐based strategy allowed for systematic optimization of critical chromatographic parameters, including MP composition, flow rate, and detection wavelength, resulting in a highly sensitive and robust method. Using a CCD, the influence of MP ratio and pH on key chromatographic responses, capacity factor, void time, retention time, asymmetry factor, and number of theoretical plates was evaluated. Optimal performance was achieved at an MP ratio of 65:35 (acetonitrile: phosphate buffer) and pH 6.75, yielding a short retention time (2.41 min), high column efficiency (8706 theoretical plates), and acceptable peak symmetry (asymmetry factor 0.95). These results demonstrate that the method is suitable for routine analysis of KBrO_3_ in bread.

Method validation was conducted according to ICH Q2(R1) guidelines and confirmed the method’s accuracy, precision, linearity, specificity, robustness, and remarkable sensitivity. Recovery studies showed high recovery rates (98.6%–99.6%), and low %RSD values (< 2%) were observed for repeatability, intraday, and interday precision, indicating method reliability under different experimental conditions. The LOD (0.015 μg/mL) and LOQ (0.04 μg/mL) were below the safety limits established by the FDA and WHO (0.02 mg/kg), allowing trace‐level detection. The absence of interfering peaks from bread matrices further confirmed the method’s specificity, making it suitable for food safety applications.

The developed and validated HPLC method was successfully applied to analyze 15 bread samples collected from various subcities. The results revealed the presence of KBrO_3_ in all samples, with concentrations ranging from 4.5 to 9.15 mg/kg, significantly exceeding the WHO and FDA safety threshold of 0.02 mg/kg.

The findings of the present study are consistent with reports from other countries, including Bangladesh, Nigeria, Tanzania, Libya, and Ethiopia, where KBrO_3_ has been detected in bread products exceeding permissible limits. In Bangladesh, a study analyzing 39 bread samples reported KBrO_3_ levels as high as 5.53 mg/kg, exceeding the FDA’s permissible limit of 0.02 mg/kg [17]. In Nigeria, a study assessing nine bread samples found all tested positive for KBrO_3_, with concentrations ranging from 2.05 ± 0.01 mg/kg to 66.22 ± 0.01 mg/kg [18], while another study in the same country showed levels between 3.85 ± 0.01 and 11.13 ± 0.00 mg/kg [19]. Similarly, research in Tanzania analyzed 21 bread samples from the Mwanza and Kagera Regions and found KBrO_3_ levels ranging from 1.02 to 4.85 mg/kg, all above the FDA’s permissible limit [20]. In Libya, a study conducted in Benghazi reported concentrations ranging from 9 to 60 mg/kg in 29 bread samples [21]. Within Ethiopia, studies conducted in five bakeries in Addis Ababa reported mean KBrO_3_ levels ranging from 5.62 ± 0.07 mg/kg to 9.97 ± 0.09 mg/kg (2). These results indicate that the KBrO_3_ levels detected in bread samples from Mekelle fall within the broader range reported nationally and internationally but remain far above the safe consumption thresholds established by WHO and FDA.

The significance of this study lies in providing a validated, sensitive, and robust analytical method for routine monitoring of KBrO_3_ in bread. The findings highlight the need for continuous surveillance and stricter enforcement of food safety regulations to protect consumers from exposure to harmful additives. Overall, this study contributes valuable data to the growing body of evidence regarding KBrO_3_ contamination in commercially available bread and demonstrates the practical application of a QbD‐based HPLC method in food safety assessment.

## 5. Strengths and Limitations of the Study

A major strength of this study is the systematic use of a QbD approach, which enabled the development and validation of a highly sensitive, accurate, and reliable HPLC method for KBrO_3_ detection and quantification. The use of stratified sampling across multiple subcities ensured representative coverage of bread products in Mekelle. Additionally, the use of validated statistical tools (Design‐Expert software and ANOVA) strengthened the reliability of the results. Furthermore, the method’s very low LOD and LOQ values provide strong potential for routine food safety monitoring.

Despite these strengths, the study has certain limitations. Although the sample was representative, the study was restricted to a single city, which may limit the generalizability of the findings to other regions of Ethiopia.

## 6. Conclusion

A robust and sensitive HPLC method for KBrO_3_ determination in bread was developed using a QbD‐based approach. The method was optimized using CCD and validated successfully following ICH guidelines, indicating its suitability for routine analysis and regulatory compliance. The method developed here provides a valuable analytical tool for monitoring bromate residues in baked products, supporting both public health protection and regulatory standards. Furthermore, regular surveillance of KBrO_3_ in food products is highly recommended, as this study revealed levels exceeding internationally accepted safety limits.

## Funding

This work was supported by NORAD under grant number RAD/External/SS045/2024, with a duration from December 2024 to December 2025.

## Conflicts of Interest

The authors declare no conflicts of interest.

## Data Availability

The data that support the findings of this study are available from the corresponding author upon reasonable request.
